# Association between omentin-1, adiponectin and bone health under consideration of osteoprotegerin as possible mediator

**DOI:** 10.1007/s40618-016-0544-3

**Published:** 2016-09-10

**Authors:** J. Menzel, R. Di Giuseppe, R. Biemann, K. Aleksandrova, O. Kuxhaus, C. Wittenbecher, A. Fritsche, M. B. Schulze, B. Isermann, H. Boeing, C. Weikert

**Affiliations:** 1 Department of Molecular Epidemiology, German Institute of Human Nutrition Potsdam-Rehbruecke, Arthur-Scheunert-Allee 114-116, 14558 Nuthetal, Germany; 2 Institute for Social Medicine, Epidemiology and Health Economics, Charité University Medical Center, Berlin, Germany; 3 German Center for Diabetes Research (DZD), München-Neuherberg, Germany; 4 Institute of Epidemiology, Christian-Albrechts University Kiel, Kiel, Germany; 5 Institute for Clinical Chemistry and Pathobiochemistry, Otto-von-Guericke University Magdeburg, Magdeburg, Germany; 6 Nutrition, Immunity and Metabolism Start-up Lab, Department of Epidemiology, German Institute of Human Nutrition Potsdam-Rehbruecke, Nuthetal, Germany; 7 Department of Epidemiology, German Institute of Human Nutrition Potsdam-Rehbruecke, Nuthetal, Germany; 8 Division of Endocrinology, Diabetology, Nephrology, Vascular Disease and Clinical Chemistry, Department of Internal Medicine, University of Tübingen, Tübingen, Germany; 9 Department of Food Safety, Federal Institute for Risk Assessment, Berlin, Germany

**Keywords:** Intelectin-1, Osteoclastogenesis inhibitory factor, Bone mineral density, Broadband ultrasound attenuation

## Abstract

**Purpose:**

Several studies implicated a crosstalk between bone and fat in the pathogenesis of osteoporosis. Few studies indicated an association between adiponectin and omentin-1 on the bone remodeling process and bone mineral density, and suggested osteoprotegerin (OPG) as a mediator of this relationship. However, only limited evidence on this relationship is available in humans. Therefore, this study aimed to investigate the association between omentin-1, adiponectin and broadband ultrasound attenuation (BUA) in peri-/premenopausal and postmenopausal women, and to assess the role of OPG as a possible mediator.

**Methods:**

Data from the German population-based EPIC-Potsdam cohort comprising 637 women were analyzed. Multivariable-adjusted ANCOVA including age, BMI, waist circumference, smoking status, education, physical activity, adiponectin or omentin-1 and hormone use was used to investigate potential relationships between the adipokines and BUA levels. A mediation analysis assessed the mediating effect of OPG on the association of BUA and omentin-1 levels.

**Results:**

Peri-/premenopausal women had higher BUA levels (112.5 ± 10.1 dB/MHz), compared to postmenopausal women (106.3 ± 10.0 dB/MHz). In peri-/premenopausal women neither adiponectin nor omentin-1 was significantly associated with BUA. In postmenopausal women, adiponectin was not associated with BUA, but 10 % increase in the omentin-1 concentration was significantly associated with 0.44 dB/MHz lower BUA levels (*p* = 0.01). Omentin-1 was positively associated with OPG (*p* = 0.02); however, OPG was not significantly related to BUA (*p* = 0.62).

**Conclusion:**

Our study provides evidence for an inverse association between circulating omentin-1 and BUA levels in postmenopausal women. However, the present findings do not support a mediating effect of OPG in the adipose tissue–bone pathway.

**Electronic supplementary material:**

The online version of this article (doi:10.1007/s40618-016-0544-3) contains supplementary material, which is available to authorized users.

## Introduction

Osteoporosis is a systemic skeletal disease characterized by reduced bone mass and micro-architectural deterioration of bone tissue with a consequent increase in bone fragility, susceptibility to fractures and reduced quality of life [1]. In women, an accelerated loss of bone mineral density (BMD) occurs after menopause. Many factors, including low BMI [2], decreased physical activity [3] and smoking [4], contribute to this bone loss. Recently, it has been suggested that adipocyte-dependent hormonal factors may play an important role in bone health [5, 6]. In particular, omentin-1 and adiponectin may act as novel determinants of BMD.

Adiponectin is an adipocyte-secreted adipokine inversely related to adiposity. Adiponectin has been suggested to exert anti-inflammatory, antiatherogenic, and insulin-sensitizing effects [7]. Notably, adiponectin and its receptors have been found to be produced by human bone-forming cells, suggesting that adiponectin may link adipose tissue to BMD [8]. A recent meta-analysis noticed an inverse association between adiponectin and BMD [9]. The association between adiponectin and the remodeling process, i.e., the removal of mineralized bones by osteoclasts followed by the formation of new bone through the osteoblasts, has also been studied in experimental in vitro and in vivo studies [10], however, showing conflicting results of adiponectin on bone metabolism. On the one hand, adiponectin was suggested to induce osteoblast proliferation and differentiation [11, 12]. On the other hand, it has been demonstrated that adiponectin promotes increased osteoclast formation indirectly by stimulating receptor activator for nuclear factor κB ligand (RANKL) and by inhibition of osteoprotegerin (OPG) production [13, 14]. OPG and RANKL are both secreted by osteoblasts. OPG belongs to the TNF-receptor superfamily and is part of a newly delineated cytokine system, and acts as a decoy receptor, binding to RANKL, preventing the activation of precursor cells, and ultimately inhibiting bone resorption [15]. RANKL, binds its receptor (RANK) settled on osteoclast precursor, thus leading to osteoclast differentiation [15]. Thus a systemic effect of adiponectin on BMD remains unclear.

Omentin-1 was discovered from a human omental fat cDNA library and is mainly expressed in visceral adipose tissue [16]. Recent studies have shown that omentin-1 is inversely related to BMI and waist circumference [17]. Moreover, positive associations with adiponectin and high density lipoprotein cholesterol were reported [17].

To date, only a few cross-sectional studies investigated the association between circulating omentin-1 and BMD in apparently healthy people showing inconsistent results [18–21]. In an experimental study high omentin-1 concentrations restored BMD, via RANKL inhibition and stimulation of OPG [22].

The present study aimed to investigate the relationship between omentin-1, adiponectin and broadband ultrasound attenuation (BUA), as one parameter reflecting bone health, in peri-/premenopausal and postmenopausal women, respectively. Furthermore, we examined whether the association between omentin-1 and BUA or adiponectin and BUA was mediated by OPG.

## Methods

### Study population

The European Prospective Investigation into Cancer and Nutrition (EPIC)-Potsdam Study is a prospective cohort study. Between 1994 and 1998, 10,904 men and 16,644 women aged 35–64 years were recruited from the general population of Potsdam and surrounding communities [23]. The recruitment process was based on residents’ registration offices [23]. In women (*n* = 9711), from 1996 until the end of the recruitment phase, quantitative ultrasound measurements (QUS) were part of the baseline examination.

Omentin-1, adiponectin and OPG concentrations were measured in a random subsample of 929 out of the 9711 women with already measured BUA levels. For the present study, we excluded 12 participants due to age at baseline below 35 years, 232 due to undefined menopausal status, i.e., no clear classification of women to “peri-/premenopausal”, “postmenopausal” or “surgical menopausal” status. Moreover, we excluded women with surgical menopause (*n* = 38), postmenopausal women taking oral contraceptives (*n* = 2) or with missing covariates data (*n* = 8). The final study population consisted of 637 women (388 peri-/premenopausal and 249 postmenopausal).

### Quantitative ultrasound measurement

QUS were performed by trained personnel on the right os calcis using Achilles Plus Ultrasound Densitometer (Lunar Corporation, Madison, WI 53713, USA) according to manufacturer instructions. BUA was measured in decibel/megahertz (dB/MHz), predefined as the slope of the signal attenuation versus the frequency curve in the usually measured range of 0.1–1 MHz [24]. In a sub-study, 11 women were measured 10 times within 3 weeks and a within-person variation coefficient of 1.47 % of BUA was observed [25].

### Blood collection and laboratory analysis

A total of 30 ml of venous blood was collected at baseline from participants at the Potsdam study center. Levels of adiponectin, omentin-1 and OPG were determined in citrate plasma. Biomarker concentrations were multiplied by 1.16 in order to obtain levels for citrate plasma samples comparable to levels obtainable from EDTA plasma [26]. Adiponectin was quantified at the Department of Internal Medicine, University of Tübingen, with an enzyme-linked immunosorbent assay from Linco Research, St Charles, Missouri, with intra-assay and inter-assay coefficients of variation (CVs) between, 0.1–6.2 and 5.0 %, respectively. Plasma concentrations of omentin-1 and OPG were measured at the Institute of Clinical Chemistry, University Magdeburg. Omentin-1 was determined by a sandwich ELISA (Biovendor, Brno, Czech Republic) with intra-assay CVs between 3.2 and 4.1 %, inter-assay CVs between 4.4 and 4.8 % and a limit of detection of 0.5 ng/ml, according to manufacturer. We previously reported excellent reliability of single measurements of omentin-1 indicated by an intraclass correlation coefficient of 0.83 (95 %-CI 0.78–0.87) [27]. OPG concentrations were quantified at the Institute of Clinical Chemistry, University of Magdeburg, using a sandwich ELISA (Biovendor, Brno, Czech Republic) with intra-assay CVs between 2.9 and 4.9 % and inter-assay CVs between 1.7 and 9.0 % and limit of detection of 0.03 pmol/l.

### Assessment of lifestyle characteristic, dietary intake and other covariates

At baseline, self-administered questionnaires on nutrition and lifestyle were filled out and computer-based interviews on lifestyle and medical histories were conducted [28]. Calibrated baseline EPIC-Germany physical activity data was used. Briefly, the physical activity levels calculation was based on a comprehensive physical activity questionnaire and objectively measured acceleration counts [3, 29]. Trained and quality personnel took the anthropometric measurements with participants wearing light underwear and no shoes, weight was measured with electronic digital scales, accurate to 100 g (Soehnle, type 7720/23, Murrhardt, Germany) and the height to the nearest 0.1 cm by using a flexible anthropometer. BMI was calculated as body weight (kg) divided by squared height (m^2^). Menopausal status was assessed according to self-reported information about menstrual status and history. Self-reported medication over the past 4 weeks prior to study enrolment was used to identify users of oral contraceptive, hormone replacement therapy (HRT).

### Statistical analysis

All analyses were performed separately for peri-/premenopausal (*n* = 388) and postmenopausal (*n* = 249) women. Normally distributed variables were reported as mean and standard derivation. Right skewed variables (omentin-1, adiponectin, osteoprotegerin) were reported as median and interquartile range, and log transformed for the analyses. Categorical variables were reported as percentage (smoking status, educational level, oral contraceptive and HRT use). For comparison of the characteristics between peri-/premenopausal women and postmenopausal women a Chi-square test for categorical variables and a Student’s *t* test or Mann–Whitney *U* test for continuous variables was used. Correlations between adipokines, BUA and OPG were assessed using Spearman age-adjusted partial correlation coefficients. Multivariable linear regression models were used to estimate the associations between the adipokines, adiponectin and omentin-1, with BUA, adjusted for age (continuous), BMI (continuous), waist circumference (continuous), smoking status (non-smoker, ex-Smoker <5 years, ex-Smoker ≥5 years, smoker <20 cigarettes/day, smoker ≥20 cigarettes/day), educational level (unskilled or skilled, technical college, university degree), physical activity (continuous), use of oral contraceptive (yes/no) and HRT (yes/no), respectively, for peri-/premenopausal and postmenopausal women. The models were additionally mutually adjusted for each adipokine (log-transformed omentin-1 or log-transformed adiponectin). Multivariable-adjusted analysis of covariance (ANCOVA) was used to assess the relationship between omentin-1 and BUA according to menopausal status-specific quartiles of omentin-1. Same analysis was performed according menopausal status-specific quartiles of adiponectin. To examine whether the association between omentin-1 and BUA was mediated by OPG, three fully adjusted linear regressions models were fitted based on the conventional steps outlined by Baron and Kenny [30]. The first model investigated the association between the mediator and the independent variable (path a). The second model determined the association between the dependent variable and independent variable (path c). The third model examined the association between the dependent variable and both independent variable and mediator included in the final model (path b and c’) [30]. The mediation model decomposes the total effect of independent variable on dependent variable (path c), into two parts: the indirect effect of independent variable on dependent variable via mediator, quantified by the product of the β-coefficients of path a and path b, and the direct effect of independent variable on dependent variable with the effect of the possible mediator removed, quantified by the path c′ (c = ab + c′) [31]. A bootstrapping analysis (1000 bootstrap samples, sampling rate 80 %) was used to estimate the mean size (95 %-CI) of total, direct and indirect effect, and further to test the statistical significance of the indirect effect [32]. The variance accounted for (VAF) determines the size of the indirect effect in relation to the total effect (VAF > 80 %: full mediation; 80 % > VAF > 20 %: partial mediation; VAF < 20 %: no mediation) [33]. A sensitivity analysis, performed in 243 participants noticed no changes in results with additionally adjustment of vitamin D, estradiol and sex hormone binding globulin (data not shown). A *p* value <0.05 was considered to be statistically significant. All statistical analyses were performed using SAS software, version 9.4 (SAS institute, Cary, N.C., USA).

## Results

Table 1 shows the basic characteristics of the 637 women, stratified by menopausal status. Compared to postmenopausal women (*n* = 249), peri-/premenopausal women (*n* = 388) were younger (*p* < 0.0001), had higher BUA (*p* < 0.0001), lower omentin-1 (*p* < 0.0001), OPG (*p* < 0.0001) and adiponectin levels (*p* = 0.0002).

**Table 1:** Characteristics of the study population according to menopausal status (EPIC-Potsdam Study, women, *n* = 637)

	Peri-/premenopausal women (*n* = 388)	Postmenopausal women (*n* = 249)	*p* value
BUA (dB/MHz)	112.5 ± 10.1	106.3 ± 10.0	<0.0001
Age (years)	41.2 ± 4.6	59.0 ± 3.6	<0.0001
BMI (kg/m^2^)	24.3 ± 4.5	27.3 ± 4.8	<0.0001
Waist circumference (cm)	77.2 ± 11.4	85.2 ± 11.3	<0.0001
Physical activity (counts/min/day)	41.0 ± 5.2	32.0 ± 5.1	<0.0001
Smoking status (%)			<0.0001
Non-smoker	44.3	64.3	
Ex-smoker <5 years	20.9	17.7	
Ex-smoker ≥5 years	9.5	5.6	
Smoker <20 cigarettes/day	20.6	10.4	
Smoker ≥20 cigarettes/day	4.9	2.0	
Educational level (%)			<0.0001
Unskilled or skilled	31.2	42.6	
Technical college	26.0	36.1	
University degree	42.8	21.3	
Oral contraceptive intake (%)	34.5	–	<0.0001
Hormone replacement therapy (%)	–	24.1	<0.0001
Adiponectin (µg/ml)	8.7 (6.6–12.0)	10.4 (7.6–13.6)	0.0002
Omentin-1 (ng/ml)	368.3 (303.3–439.1)	452.4 (374.7–546.7)	<0.0001
Osteoprotegerin (pmol/l)	4.3 (3.6–5.3)	5.1 (4.3–5.7)	<0.0001

Variables are expressed as percentage, or mean ± standard deviation, or median (interquartile range)

We noticed no relevant correlations between adiponectin and BUA or OPG in peri-/premenopausal women as well as in postmenopausal women (Online Resource 1). In line, multivariable-adjusted linear regression models detected no associations between adiponectin and BUA (Table 2), as well as between adiponectin and OPG levels in both peri-/premenopausal or postmenopausal women.

**Table 2:** Quartiles of omentin-1 and adiponectin with adjusted BUA values according to menopausal status

	*n*	Omentin-1 (ng/ml)	BUA (dB/MHz)^a,b^	*p* for trend
		Median (IQR)	Adjusted mean (95 %-CI)	
Peri-/premenopausal women (*n* = 388)				
Q1	97	256.4 (230.8, 285.4)	111.0 CI (108.8, 113.2)	0.4
Q2	97	334.1 (321.3, 350.3)	111.0 CI (108.8, 113.2)	
Q3	97	395.6 (384.0, 419.9)	112.9 CI (110.6, 115.2)	
Q4	97	494.2 (460.5, 568.4)	111.7 CI (109.4, 114.1)	

	*n*	Omentin-1 (ng/ml)	BUA (dB/MHz)^a,d^	*p* for trend
		Median (IQR)	Adjusted mean (95 %-CI)	
Postmenopausal women (*n* = 249)				
Q1	62	322.9 (284.2, 342.2)	107.0 CI (103.8, 110.2)	0.02
Q2	63	417.6 (394.4, 436.2)	106.9 CI (103.7, 110.2)	
Q3	62	495.9 (472.1, 520.8)	106.2 CI (103.2, 109.2)	
Q4	62	614.2 (590.4, 692.5)	103.0 CI (99.6, 106.4)	

	*n*	Adiponectin (µg/ml)	BUA (dB/MHz)^b,c^	*p* for trend
		Median (IQR)	Adjusted mean (95 %-CI)	
Peri-/premenopausal women (*n* = 388)				
Q1	97	5.42 (4.46, 5.93)	110.6 CI (108.3, 112.9)	0.9
Q2	97	7.82 (7.17, 8.29)	112.9 CI (110.7, 115.0)	
Q3	97	10.30 (9.48, 11.10)	112.5 CI (110.2, 114.7)	
Q4	97	14.31 (13.07, 15.90)	110.5 CI (108.2, 112.8)	

	*n*	Adiponectin (µg/ml)	BUA (dB/MHz)^c,d^	*p* for trend
		Median (IQR)	Adjusted mean (95 %-CI)	
Postmenopausal women (*n* = 249)				
Q1	62	5.56 (5.04, 6.67)	106.8 CI (103.6, 110.0)	0.3
Q2	62	9.14 (8.44, 9.70)	106.0 CI (102.7, 109.3)	
Q3	63	11.65 (10.98, 12.41)	106.9 CI (103.7, 110.1)	
Q4	62	16.37 (14.29, 17.82)	104.2 CI (101.0, 107.5)	

Adjusted for age, waist circumference, BMI, smoking status, education, physical activity

^a^Additionally adjusted for adiponectin (log transformed)

^b^Additionally adjusted for oral contraceptive use

^c^Additionally adjusted for omentin-1 (log transformed)

^d^Additionally adjusted for hormone replacement therapy

We observed no association between omentin-1 and BUA in peri-/premenopausal women (Table 2). However, in postmenopausal women, omentin-1 was inversely related to BUA levels even after adjustment for age, BMI, waist circumference, smoking status, educational attainment, use of HRT and log-transformed adiponectin levels. In particular, a 10 % increase in omentin-1 levels was significantly associated with 0.44 dB/MHz lower BUA (*p* = 0.01) (107.0 95 %-CI 103.8–110.2 dB/MHz versus 103.0 95 %-CI 99.6–106.4 dB/MHz for the highest versus the lowest quartile of omentin-1; Table 2).

**Fig. 1:**
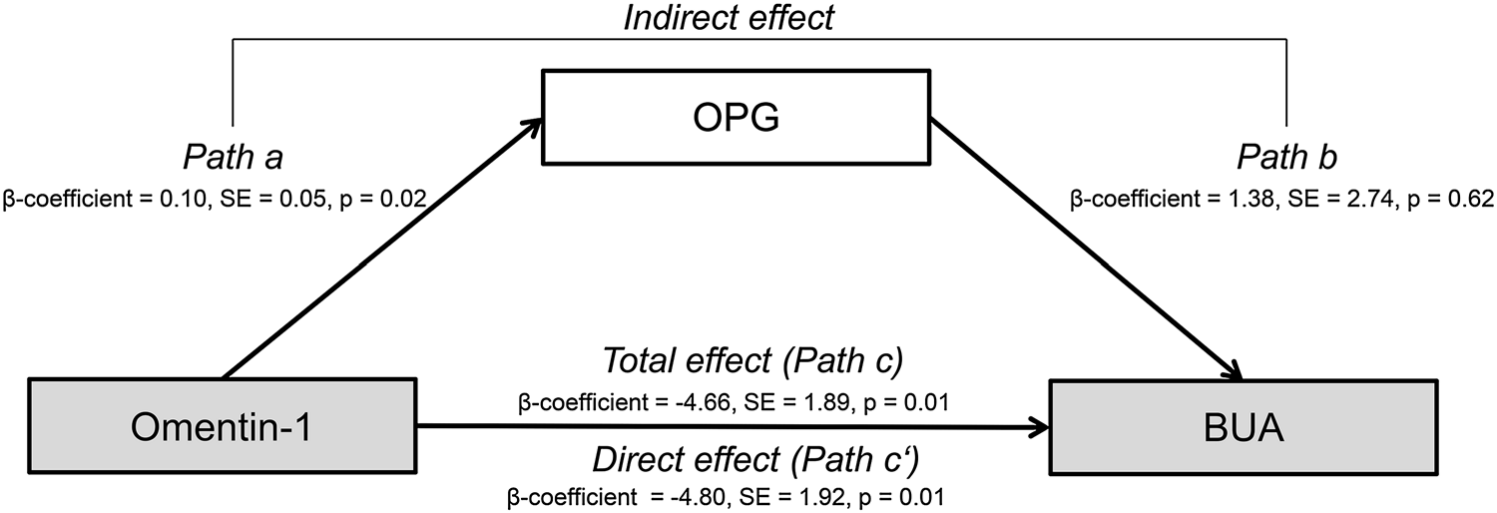
OPG (log transformed) mediation model of the relationship between omentin-1 (log transformed) and BUA in postmenopausal women (*n* = 249). Mediation model decomposes the total effect of omentin-1 on BUA (path c), into indirect effect of omentin-1 on BUA via mediator OPG, quantified by the product of the β-coefficients of path a and path b, and the direct effect of omentin-1 on BUA, when the effect of the possible mediator was removed, quantified by the path c′; adjusted for age, BMI, waist circumference, physical activity, smoking, education, hormone replacement therapy, log-transformed adiponectin; BUA: broadband ultrasound attenuation, *OPG* osteoprotegerin, *SE* standard error

Therefore, we tested the mediating effect of OPG on the association between BUA and omentin-1 levels in postmenopausal women only. As shown in Fig. 1, a 10 % increase in omentin-1 was associated with 1.0 % higher OPG levels (*p* = 0.02), whereas omentin-1 was negatively associated with BUA levels. When omentin-1 and OPG were simultaneously included in the fully adjusted model, OPG was not significantly associated with BUA levels (*p* = 0.62) (Fig. 1). Bootstrapping analysis showed a statistically significant indirect effect of omentin-1 on BUA via OPG (β-coefficient mean bootstrap 0.13, 95 %-CI bootstrap 0.12-0.14; Table 3). However, the VAF in mediation model was 3 %, suggesting lack of mediation effect of OPG on the association between omentin-1 and BUA levels (Table 3).

**Table 3:** Decomposition of the total effect of omentin-1 (log transformed) on BUA in postmenopausal women (*n* = 249) into an indirect effect mediated by OPG (log transformed) and a non-mediated direct effect

	Mean bootstrap	95 %-CI bootstrap
Total effect (β-coefficient)	−4.63	−4.69 to −4.57
Direct effect (β-coefficient)	−4.76	−4.82 to −4.70
Indirect effect (β-coefficient)	0.13	0.12 to 0.14
VAF (%)	3.00	2.77 to 3.23

Mean and 95 %-CI were estimated by bootstrapping analysis (1000 bootstrap samples, sampling rate 80 %)

*VAF* variance accounted for mediation

## Discussion

In the present study we investigated the relationships between the adipokines, adiponectin and omentin-1, and BUA and examined the role of OPG as a possible mediator in these associations in peri-/pre- and postmenopausal women. Adiponectin was not related to BUA levels in both peri-/pre- and postmenopausal women; whereas omentin-1 was significantly negatively related to BUA levels in postmenopausal women only. Contrary to our hypothesis, this association was not mediated by OPG levels.

Results from a meta-analysis of observational studies suggest an inverse relationship between adiponectin and BMD, with five studies showing a negative association and five others showing no association [9]. However, in line with our findings, also other studies failed to observe an association between adiponectin and BMD levels [34–37].

Accordingly, results from in vitro and in vivo studies have shown conflicting results. On the one hand, adiponectin was suggested to induce osteoclast formation via stimulation of RANKL and inhibition of OPG production [13, 14]; on the other hand, adiponectin was shown to induce osteoblast proliferation and differentiation [12], and to increase bone mass via osteoclastogenesis suppression and osteoblastogenesis activation [11]. Yet, the reason for such discrepant findings is unclear, but it is conceivable that isolated cell culture models and animal models are limited in their capability to emulate complex processes, like possible adiponectin-dependent counter-regulatory mechanisms. Therefore, the biological action of adiponectin in bone biology still needs to be elucidated.

To date, only a few studies investigated the relationship between the novel adipokine omentin-1 and BMD in apparently healthy people. As shown by Li et al. omentin-1 was negatively correlated with BMD of the total body and lumbar spine in Chinese men; however, this correlation lost statistical significance after adjustment of age, BMI and fat body mass [20]. Moreover, a negative correlation was found between omentin-1 and BMD at femoral neck, total hip and ward’s triangle, albeit not statistically significant. In line with our results in postmenopausal women Tohidi et al. observed a significant inverse association between omentin-1 and BMD [19] and the study of Zhang et al. supports this inverse association, though this association was not statistically significant [21]. In contrast, Wang et al. [18] observed an inverse relationship between omentin-1 and BMD in premenopausal, but not in postmenopausal women.

In vitro and in vivo studies suggest that omentin-1 may play a protective role in both bone remodeling process and BMD. In co-culture systems of osteoblast and osteoclast precursors, omentin-1 was shown to reduce osteoclast formation via OPG and to inhibit RANKL production in osteoblasts [22]. Second, omentin-1 treatment significantly enhanced BMD in ovariectomized mice (a widely used mice model for postmenopausal women [38]) accompanied by higher serum OPG and lower RANKL levels, suggesting a bone-sparing effect of omentin-1 via OPG and RANKL [22].

The current study supports a positive association between omentin-1 and OPG in postmenopausal women, in line with Xie and colleagues findings [22]. However, we observed no association between OPG and BUA levels, which questions a mediating effect of OPG in the adipose tissue–bone crosstalk. Nonetheless, experimental studies indicate an OPG-dependent bone remodeling mechanism, whereas human studies have provided controversial results regarding the relationship of OPG on bone remodeling marker and BMD [39–42]. The reason for this controversy might be explained by the fact that rather than OPG, the OPG/RANKL ratio most likely influences the static measure of BMD [43], thus maintaining an appropriate balance of bone remodeling. Therefore, further studies are required to explore RANKL and OPG/RANKL ratio as potential mediators in the adipose tissue–bone association.

Overall, the higher omentin-1 and lower BUA levels observed in postmenopausal women might be due to a physiological compensation and adaptation to bone loss. Even if we observed an inverse association between omentin-1 and BUA levels, it cannot be ruled out that omentin-1 may attenuate the stronger bone removal in these women. However, the cross-sectional design of the present study does not allow for causal inference, therefore we cannot clarify whether the observed omentin-1 concentrations are pathological or compensatory. Furthermore, because the present study comprises middle-aged Caucasian women, these findings may not be generalizable to other ethnic or age groups.

Additional limitations of the present study deserve to be mentioned. First, BUA, derived from QUS measurements on the right os calcis only, was used as a proxy of BMD measures, commonly measured with dual energy X-ray absorptiometry technique. Yet, BUA has been validated satisfactorily several times against BMD [44], thus representing a non-invasive valid, inexpensive, easy, and quick alternative measure for BMD. Furthermore, the present findings are based on one single baseline measurements of omentin-1 and adiponectin levels. However, we observed excellent reliability of omentin-1 measurements within individuals over time [27]. Adiponectin level have also been shown to be stable over time [45].

Strengths of our study include the population-based sample with suitable comparison between peri-/premenopausal and postmenopausal women, and the availability of high-quality data as a result of the standardized procedures enabling us to adjust for the most important potential confounders. However, other important factors that may influence the relation between adipokines and BUA such as vitamin D or sexual hormones have not been measured in all participants of the study population.

In conclusion, this study shows no association between adiponectin and BUA levels. However, higher plasma omentin-1 levels were associated with lower BUA levels in postmenopausal women. We did not find evidence of a mediating effect of OPG in the association between omentin-1 and BUA levels.

## Electronic supplementary material

Below is the link to the electronic supplementary material.

Online Resource 1 (PDF 13 kb)
