# Supplementary material for: Association between omentin-1, adiponectin and bone health under consideration of osteoprotegerin as possible mediator
Source: J Endocrinol Invest. 2016 Sep 10;39(11):1347–55. doi: 10.1007/s40618-016-0544-3 (PMC5069301; doi:10.1007/s40618-016-0544-3)
Supplement: Supplementary file 1 — Online Resource 1 (PDF 13 kb) [file 40618_2016_544_MOESM1_ESM.pdf]

Association between omentin-1, adiponectin and bone health under consideration of osteoprotegerin as possible mediator

Journal of Endocrinological Investigation

Juliane Menzel, MSc[1,2,3]; Romina di Giuseppe, PhD, MSc[4]; Ronald Biemann, PhD[5]; Krasimira Aleksandrova, PhD, MPH[6,7]; Olga Kuxhaus[1]; Clemens Wittenbecher, MSc[1,3]; Andreas Fritsche, MD[3,8]; Matthias B Schulze, DrPH[1,3]; Berend Isermann, MD[5]; Heiner Boeing, PhD, MSc[7]; Cornelia Weikert, MD, MPH[2,9]

[1] Department of Molecular Epidemiology, German Institute of Human Nutrition Potsdam–Rehbruecke, Nuthetal, Germany

[2] Institute for Social Medicine, Epidemiology and Health Economics, Charité University Medical Center, Berlin, Germany

[3] German Center for Diabetes Research (DZD), München-Neuherberg, Germany

[4] Institute of Epidemiology, Christian-Albrechts University Kiel, Kiel, Germany

[5] Institute for Clinical Chemistry and Pathobiochemistry, Otto-von-Guericke University Magdeburg, Magdeburg, Germany

[6] Nutrition, Immunity and Metabolism Start-up Lab, Department of Epidemiology, German Institute of Human Nutrition Potsdam-Rehbruecke, Nuthetal, Germany

[7] Department of Epidemiology, German Institute of Human Nutrition Potsdam-Rehbruecke, Nuthetal, Germany

[8] Department of Internal Medicine, Division of Endocrinology, Diabetology, Nephrology, Vascular Disease and Clinical Chemistry, University of Tübingen, Tübingen, Germany

[9] Federal Institute for Risk Assessment, Department of Food Safety, Berlin, Germany

Corresponding author: Juliane Menzel (Juliane.Menzel@dife.de)

Online Resource 1 Partial spearman correlations between BUA, OPG, omentin-1 and adiponectin in peri/premenopausal and postmenopausal women

	Peri-/premenopausal women (n=388)				Postmenopausal women (n=249)			
	**BUA**	**OPG**	**Omentin-1**	**Adiponectin**	**BUA**	**OPG**	**Omentin-1**	**Adiponectin**
**BUA**	—	-0.03 (0.50)	0.01 (0.78)	-0.08 (0.12)	—	-0.002 (0.98)	-0.17 (0.007)	-0.11 (0.09)
**OPG**		—	-0.13 (0.01)	0.07 (0.20)		—	0.21 (0.0007)	0.02 (0.72)
**Omentin-1**			—	0.29 (<0.0001)			—	0.15 (0.02)
**Adiponectin**				—				—

Expressed as ρ (p-value), adjusted for age
